# Renal Involvement in Preeclampsia: Similarities to VEGF Ablation Therapy

**DOI:** 10.1155/2011/176973

**Published:** 2010-12-23

**Authors:** Janina Müller-Deile, Mario Schiffer

**Affiliations:** Division of Nephrology and Hypertension, Department of Medicine, IFB-TX Hannover, Hannover Medical School, Carl-Neuberg-Straße 1, 30625 Hannover, Germany

## Abstract

Glomerular VEGF expression is critical for the maintenance and function of an intact filtration barrier. Alterations in glomerular VEGF bioavailability result in endothelial as well as in podocyte damage. Renal involvement in preeclampsia includes proteinuria, podocyturia, elevated blood pressure, edema, glomerular capillary endotheliosis, and thrombotic microangiopathy. At least the renal signs, symptoms, and other evidence can sufficiently be explained by reduced VEGF levels. The aim of this paper was to summarize our pathophysiological understanding of the renal involvement of preeclampsia and point out similarities to the renal side effects of VEGF-ablation therapy.

## 1. Introduction

The renal involvement in preeclampsia is characterized by hypertension and proteinuria that have to present after the twentieth week of gestation to fulfil the clinical diagnosis. It is also associated with edema and hyperuricemia [1]. Glomerular capillary endotheliosis and thrombotic microangiopathy (TMA) are more severe findings. The pathophysiology of these phenomena is explained by excess placental soluble fms like tyrosin kinase-1 (sFlt-1) that binds circulating vascular endothelial growth factor (VEGF) and placenta growth factor (PlGF) and prevents their interaction with endothelial cell-surface receptors. sFlt-1 is also known as soluble VEGF receptor-1 (sVEGFR-1). Other abbreviations for PlGF are PGF and PLGF. 

VEGF is a potent promoter of angiogenesis; it regulates endothelial cell function by induction of nitric oxide and vasodilatation and decreases vascular tone and blood pressure [2]. The major source of glomerular VEGF under normal conditions is the podocyte [3]. During pregnancy the placenta is the main source of sFlt-1 and PIGF. 

In 2003, Maynard et al. observed that the serum levels of both VEGF and PlGF were decreased in women with preeclampsia. However, the magnitude of decrease was less pronounced for VEGF since its serum level was not as high as PlGF, even in normal pregnancy [4]. De Vivo et al. showed that Endoglin, PlGF, and sFlt-1 might be used as markers for predicting preeclampsia and that sFlt-1 : PlGF-ratio is even more accurate [5]. Eventually measurement of antiangiogenic factors might become a surrogate for renal biopsy to establish the diagnosis.

Under pathological conditions VEGF is produced by various cancers to induce angiogenesis and supply the tumour with new blood vessels. In the last years anti-VEGF therapies that either block the extracellular binding of VEGF to its receptor (anti-VEGF antibodies) or inhibit intracellular signalling pathways of VEGF receptors (receptor tyrosine kinase inhibitors) have become an innovative target in the treatment of these cancers.

Since the only receptor for PIGF is VEGFR-1, antiangiogenetic targets blocking this receptor inhibit both VEGF signalling as well as the PLGF pathway. 

In oncology trials that antagonize VEGF using neutralizing antibodies and VEGF receptor inhibitors hypertension, proteinuria and TMA can also occur similar to the phenomena seen in preeclampsia [6, 7].

First evidence for similarities between preeclampsia and side effects of VEGF ablation therapy in the kidney emerged from studies in animals. An adenovirus-expressing sFlt-1 in rodents caused a clinical syndrome with features of preeclampsia, including glomerular endotheliosis, proteinuria, and hypertension [4]. 

In an institutional review board-approved case series Patel et al. described seven patients who developed a “preeclampsia-like syndrome” characterized by hypertension and proteinuria after starting therapy with sunitinib and sorafenib, two multityrosine kinase inhibitors interfering with VEGF signalling. The patients were identified clinically after developing edema, hypertension, proteinuria, and/or hypoalbuminemia [8]. 

In the following sections, we will highlight the renal symptoms occurring in both preeclampsia as well as VEGF ablation therapy. To accomplish this we performed a selective literature search in PubMed database using the key words “preeclampsia,” “preeclampsia AND proteinuria,” “preeclampsia AND podocyturia,” “preeclampsia AND edema,” “preeclampsia AND renal thrombotic microangiopathy,” “VEGF ablation therapy,” and “VEGF inhibition AND renal thrombotic microangiopathy.”

## 2. Proteinuria

Proteinuria is one of the essential symptoms for the clinical diagnosis of preeclampsia. Any process that induces a disturbance of the glomerular endothelium, and the glomerular basement membrane (GBM) or changes in podocyte function can lead to proteinuria. It has been shown for many renal diseases that disruptions or an imbalance of slit diaphragm proteins lead to podocyte effacement and proteinuria. Podocytes are the major source of VEGF production in the glomerulus. Podocyte-derived VEGF has well-documented paracrine functions on endothelial cells as well as autocrine functions on podocytes themselves [9–11]. However, it remains controversial which VEGF receptor is most critically involved in autocrine functions of VEGF on podocytes [12].

Proteinuria and elevations of sFlt-1 that neutralize VEGF are temporally related in preeclampsia [13] suggesting a pathophysiological role of sFlt-1 in the development of proteinuria. However, sFlt-1 levels rise before sings and symptoms of preeclampsia arise [14].

Renal tissue from autopsy material from preeclamptic women identified a reduction in nephrin and synaptopodin expression in preeclamptic glomeruli [15]. Nephrin and synaptopodin are podocyte proteins and are essential for the maintenance of podocyte functions and the integrity of the slit diaphragm. Similarly, Sugimoto et al. demonstrated that anti-VEGF antibodies and sFlt-1 caused glomerular endothelial cell detachment and hypertrophy as well as downregulation of nephrin [16].

VEGF ablation therapy by bevacizumab (a humanized monoclonal antibody that recognizes and blocks vascular endothelial growth factor A (VEGF-A)), sorafenib (a small molecular inhibitor of several tyrosine kinases), sunitinib (an oral, small-molecule, multitargeted receptor tyrosine kinase (RTK) inhibitor) and other anti-VEGF drugs are frequently complicated by mild proteinuria and hypertension. Anti-VEGF therapy has been associated with the onset of proteinuria in 23%–38% of patients with colorectal cancer and in up to 64% of patients with renal cell carcinoma [17]. Although this proteinuria was largely asymptomatic and low-grade, high-grade proteinuria and acute kidney injury have been described in some cases [6, 18].

There is a profound body of evidence that VEGF is important in maintaining glomerular endothelial cell health and healing [19] and that its absence induces proteinuria, release of procoagulant proteins, and glomerular endotheliosis [16, 20].

All these studies indicate that neutralization of circulating VEGF (either by sFlt-1 or antiangiogenetic drugs) may play an important role in the induction of proteinuria in cancer therapy and in women with preeclampsia.

## 3. Podocyturia

Glomerular podocytes are known to regulate proteinuria and proteinuria is aften accompanied by podocyturia (excretion of viable podocytes) that often accompany proteinuria. Podocyturia is a highly sensitive and specific marker for preeclampsia and we were able to demonstrate earlier together with the Mayo-Clinic that podocyturia may contribute to the development of proteinuria in preeclampsia [13].

Interestingly a statistical analysis by Aita et al. indicated a correlation between urinary podocyte number and blood pressure, but not with proteinuria [21]. Because of the significant correlation between blood pressure and the extent of podocyturia Yu et al. saw a causal role of hypertension in podocyte loss [22].

We earlier demonstrated that urinary loss of podocalyxin positive cells reflects active glomerular damage better than proteinuria (Achenbach et al. [23]) and we have reported podocyturia in patients under VEGF ablation therapy with bevacizumab or sunitinib (Müller-Deile et al. [24]). We therefore propose that podocyturia is a useful noninvasive and predictive marker of ongoing glomerular damage in women with preeclampsia and in patients undergoing VEGF ablation therapy.

## 4. Blood Pressure

In a study by Robinson et al. the inhibition of the VEGF receptor signalling pathway by cediranib (a potent inhibitor of recombinant KDR tyrosine kinase (VEGFR-2)) induced a rapid but variable rise in blood pressure in most patients within three days. Because of the rapid development of hypertension, acute inhibition of VEGF-dependent vasodilatation was suggested as explanation [25]. Hertig defined this as a “preeclampsia-like syndrome” in regard of hypertension and glomerular proteinuria, complicating the treatment with anti-VEGF agents. Quoting Hertig this syndrome is usually mild or moderate and should be treated by the addition of an antihypertensive drug while the most severe forms require the interruption of the regimen [26]. The exact etiology of hypertension in VEGF ablation therapy is unclear. There are several explanations for hypertension in consequence to reduced VEGF. The first indications that VEGF is important for blood pressure regulation came form preclinical studies where Li et al. could show that VEGFR-2 is the major mediator for the hypotensive effect of VEGF [27]. VEGF induces the release of nitric oxide (NO) and prostacyclin (PGI_2_) by endothelial cells leading to vasodilatation [28]. Therefore the inhibition of VEGF either by sFlt-1 in preeclampsia or by VEGF ablation therapy may result in elevated blood pressure. Another cause for hypertension in response to reduced VEGF may be due to decreased number of small blood vessels. Decreased density of microvessels leads to increased peripheral vascular resistance and reduced NO activity. There may also be an association between therapy-induced hypertension and proteinuria. Independent of this, VEGF can induce hypotension through an endothelial baroreceptor response [29]. Neutralization of circulating VEGF could antagonize this effect. Moreover, blocking VEGF signalling pathway may also interfere with the balance between VEGF and endothelin which is a potent vasoconstrictor [30].

In contrast to the findings above recombinant VEGF-A_121_ infusion lowered blood pressure and improved renal function in rats with placental ischemia-induced hypertension [31].

Li et al. used a virus-infused preeclampsia model that raises sFlt-1 levels and has been shown to cause renal glomerular endotheliosis. By infusing VEGF-A_121_, they could also demonstrate decreased blood pressure and proteinuria as well as improvement and reversal of the glomerular lesions [32].

## 5. Edema

Edema that can occur during angiogenesis inhibitor therapy by VEGF ablation is one of the dose-limiting toxicities for sunitinib. It may also be a direct consequence of the proteinuria [33]. Edema which is not essential for the diagnosis of preeclampsia is nevertheless a frequent phenomenon in this disease.

## 6. Glomerular Capillary Endotheliosis

Preeclampsia is associated with a characteristic glomerular lesion often described as glomerular capillary endotheliosis. In rats, the reduction in VEGF-A dosage reduces the activation of endothelial nitric oxide synthase (eNOS) and blocks the vasodilatation. This contributes to the development of systemic endotheliosis. 

Another suggestion that downregulation of VEGF signalling within the glomerulus may be involved in the renal lesion of preeclampsia is given by S. Quaggin's group: they described the development of preeclampsia-like endotheliosis and “bloodless glomeruli” in mice with podocyte-specific heterozygosity for VEGF. By 9 weeks of age, all of the podocyte-specific VEGF heterozygotes developed end-stage kidney failure due to a severe form of glomerulosclerosis with loss of differentiated podocytes and endothelial cells [34].

VEGF-A is also thought to be involved in glomerular endothelial cell fenestration [35]. In both, preeclampsia and VEGF ablation therapy reduced dosage of local VEGF-A led to decreased vasodilatation and reduced formation of fenestrae.

## 7. Glomerular Thrombotic Microangiopathy

Another parallelity between preeclampsia and VEGF ablation therapy is the risk for thrombotic events especially thrombotic microangiopathy (TMA) [36, 37]. A case of histologically documented TMA secondary to sunitinib was presented by Bollée et al. [38]. Costero et al. reported a patient who showed arterial hypertension, nephrotic syndrome, and focal segmental glomerulosclerosis (FSGS) in addition to TMA ten months after treatment with sunitinib. The syndrome disappeared six months after sunitinib withdrawal [39].

Frangié et al. reported the occurrence of TMA in association with anti-VEGF antibody treatment for metastatic renal cell carcinoma [40]. TMA was also seen in association with VEGF-Trap (a fully humanized recombinant fusion protein containing extracellular portions of VEGFR-1 and VEGFR-2) for the treatment of metastatic ovarian cancer [41]. Importantly, Eremina et al. Showed that TMA due to anti-VEGF treatment is reversible after the drug is withdrawn [42].

This course is similar to that seen in preeclampsia which typically resolves after delivery of the placenta when the source of excess sFlt-1 no longer exists [5].

For preeclampsia Zhang et al. reported a patient who developed thrombotic microangiopathy secondary to preeclampsia at 18 week of gestation [43]. Ducroz et al. found similarities between the clinical, biological, and histological features of thrombotic microangiopathy and the HELLP syndrome [44]. Interestingly, Verheul and Pinedo explained the thrombotic events by induction of apoptosis of endothelial cells due to the blockade of VEGF pathway. The lack of endothelial cell renewal would in consequence lead to exposure of the basement membrane to the circulating blood and result in platelet activation [33].

The hypothesis that external VEGF may accelerate recovery of renal microvascular injury was studied in an experimental TMA model. VEGF_121_ infusion resulted in greater recovery of the renal microvasculature in rats with TMA, and effects of VEGF were suggested to be mediated by NO [45].

These findings are well in line with the studies of Li et al. and Gilbert et al., where VEGF also reversed glomerular lesions. 

**Figure 1:**
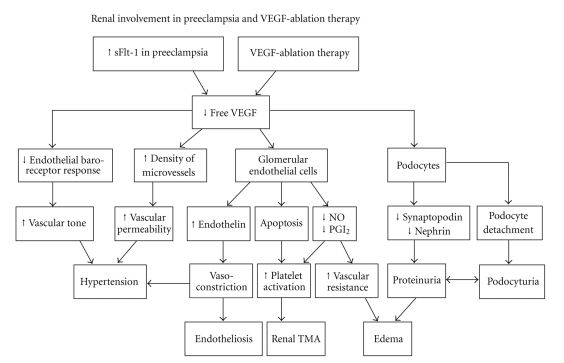
Flowchart demonstrates the renal involvement in preeclampsia and VEGF ablation therapy due to decreased levels of local and circulating VEGF. sFlt-1: soluble fms-like tyrosine kinase-1; VEGF: vascular endothelial growth factor; NO: nitric oxide; PGI_2_: prostacyclin; TMA: thrombotic microangiopathy.

So in summary, there is a large body of evidence that the renal involvement in preeclampsia and VEGF ablation therapy are explained by reduced VEGF levels as illustrated in Figure 1.

## 8. Conclusions

As increased serum levels of sFlt-1 are known to be causal factors for the pathophysiology of preeclampsia, it was interesting to look at similarities between renal involvement in preeclampsia and VEGF ablation therapy. 

A systematic literature review revealed that preeclampsia and VEGF ablation therapy can lead to proteinuria, podocyturia, elevated blood pressure, edema, glomerular capillary endotheliosis, and glomerular thrombotic microangiopathy.

These symptoms and pathologic conditions seem to have the same etiology because they all can be explained by decreased VEGF levels (Figure 1). In both preeclampsia and antiangiogenic tumour therapy the VEGF/PLGF pathway is either blocked by sFlt-1, therapeutic antibodies against the growth factors, or VEGF receptor inhibition. 

The renal analogy of preeclampsia and side effects of VEGF ablation therapy could have relevance for future diagnosis and treatment in both. 

As anti-VEGF therapy is becoming more and more frequent in cancer therapy, preeclampsia provides an example where reduced VEGF and PLGF by elevated sFlt-1 are key factors for disease development. In contrast to tumour treatment, proangiogenic therapies could repair disturbed endothelial function in preeclampsia.
